# A tryptophan-rich breakfast and exposure to light with low color temperature at night improve sleep and salivary melatonin level in Japanese students

**DOI:** 10.1186/1740-3391-11-4

**Published:** 2013-05-25

**Authors:** Kai Wada, Shota Yata, Osami Akimitsu, Milada Krejci, Teruki Noji, Miyo Nakade, Hitomi Takeuchi, Tetsuo Harada

**Affiliations:** 1 Laboratory of Environmental Physiology, Graduate School of Integrated Arts and Sciences, Kochi University, Kochi, Japan; 2 Department of Health and Physical Education, Faculty of Education, Kochi University, Kochi, Japan; 3 Department of Health Education, Faculty of Education, University of South Bohemia, České Budějovice, Czech Republic; 4 Department of Nutritional Education, Tokai Gakuen University, Miyoshi, Aichi, Japan

**Keywords:** Salivary melatonin, Tryptophan, Protein rich breakfast, Sunlight exposure, Lighting with low color temperature

## Abstract

**Background:**

Epidemiological studies in Japan have documented an association between morning type and a tryptophan-rich breakfast followed by exposure to sunlight in children. The association may be mediated by enhanced melatonin synthesis, which facilitates sleep at night. However, melatonin is inhibited by artificial light levels with high color-temperature common in Japanese homes at night. In this study, we investigated whether a combination of tryptophan-rich breakfast and light with low color-temperature at night could enhance melatonin secretion and encourage earlier sleep times.

**Methods:**

The intervention included having breakfast with protein- and vitamin B6 - rich foods and exposure to sunlight after breakfast plus exposure to incandescent light (low temperature light) at night (October-November, 2010). The participants were 94 members of a university soccer club, who were divided into 3 groups for the intervention (G1: no intervention; G2: asked to have protein-rich foods such as fermented soybeans and vitamin B6-rich foods such as bananas at breakfast and sunlight exposure after breakfast; G3: the same contents as G2 and incandescent light exposure at night). Salivary melatonin was measured around 11:00 p.m. on the day before the beginning, a mid-point and on the day before the last day a mid-point and on the last day of the 1 month intervention.

**Results:**

In G3, there was a significantly positive correlation between total hours the participants spent under incandescent light at night and the frequency of feeling sleepy during the last week (p = 0.034). The salivary melatonin concentration of G3 was significantly higher than that of G1 and G2 in combined salivary samplings at the mid-point and on the day before the last day of the 1 month intervention (p = 0.018), whereas no such significant differences were shown on the day just before the start of the intervention (p = 0.63).

**Conclusion:**

The combined intervention on breakfast, morning sunlight and evening-lighting seems to be effective for students including athletes to keep higher melatonin secretion at night which seems to induce easy onset of the night sleep and higher quality of sleep.

## Background

Tryptophan is an essential amino acid that can be absorbed exclusively from meals in humans. It is metabolized via 5-hydroxytryptamine (serotonin) to melatonin by a series of 4 enzymes in the pineal body [1,2]. Serotonin is known as a precursor to melatonin. A lack of serotonin causes depression, panic disorder, obsessive-compulsive disorder, sleep disorders and eating disorders [3] and induces aggression, anxiety/aggression-driven depression, impulsive behavior and suicidal attempts [4,5]. Serotonin thus has a strong relationship with mental health. In the past two decades, serotonin reuptake inhibitors (SSRIs) have come to be widely used for the treatment of affective disorders including depression, although [6] there are controversies whether SSRIs are effective or not for the treatment of depression in children and adolescents because of the shortage of coincident scientific evidence of SSRIs for young humans.

Exposure to sunlight in the daytime appears to trigger synthesis of serotonin in the pineal body [7]. This action is hypothesized to occur mainly in the morning hours, because the amount of tryptophan consumed with supper has neither significant effects on Morningness-Eveningness (M-E) scores nor an effect on sleep habits, as shown by another study on young Japanese children performed in 2005 [8].

Melatonin is synthesized in the pineal body of the hypothalamic area and secreted at night. Melatonin level in the serum can be well and positively correlated with that in the saliva [9-12]. Secretion of melatonin exhibits circadian rhythms and is suppressed by bright light at night [13,14]. Even room lights such as fluorescent lamps can attenuate melatonin excretion duration at night [15]. Evening lighting conditions are also said to affect circadian rhythms [16,17] and mental health in mice [18]. Tryptophan intake at breakfast is effective for the onset and offset of sleep in young children [19]. Moreover, questionnaire surveys showed that young children exposed to sunlight for more than 30 minutes after having sources of protein at breakfast are more morning-typed than those exposed for less than 30 minutes [20], and that the more young children take in vitamin B6 at breakfast, the more they exhibit morning typology [21].

Although these findings imply that morning tryptophan and vitamin B6 intake and following exposure to sunlight would promote synthesis of serotonin in the daytime and further to melatonin at night, it is difficult to test the hypothesis only with questionnaire studies. Moreover, this melatonin synthesis might be inhibited by exposure to short-wave (blue) light including light emitted from fluorescent lamps. This hypothesis cannot be tested by questionnaire work and would require an intervention field experiment. An intervention field experiment for was thus performed on university students to test the hypotheses.

## Methods

**Figure 1:**
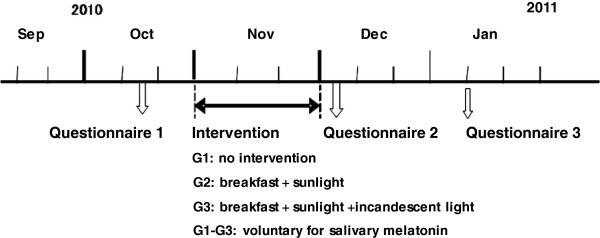
The schedules of the interventions.

The intervention program was administered to 94 subjects (male, 19-22 years old, average age: 20.33) belonging to a university soccer club. 63 subjects answered to the integrated questionnaire before the intervention period. They were divided into three groups (G1, n = 20: no intervention; G2, n = 22: asked to have protein-rich foods such as fermented soybeans and vitamin B6-rich foods such as bananas at breakfast and sunlight exposure after breakfast; G3, n = 21: the same contents as G2 and incandescent light exposure at night). This university football club includes only men. All the members used fluorescent lamps (white light) for the lighting at night. To estimate the effects of the one month interventions, integrated questionnaires were administered to all participants three times: before the start of the intervention period, immediately after the end of the intervention period, and one month after the end of the intervention period (See note on Figure 1). The questionnaires which were administered before the intervention and 1 month after the intervention consisted of the diurnal-type scale constructed by Torsvall and Åkerstedt [22], questions on sleep habits and meal habits [23], an irritation index, the General Health Questionnaire (GHQ), the Sense of Coherence (SOC) questionnaire, and FFQ (Food Frequency Questionnaire). The questionnaire just after the intervention period of 1 month consisted of self-assessment questions asking how many days during the month-long intervention period they followed the recommendations for breakfast content (the first point), sunlight exposure after breakfast (the second point) and the use of light bulbs that emit lower color temperature light at night (the third point). We made nine groups initially based on the scores of the diurnal-type scale (three groups: morning-type, middle-type, evening-type) and FFQ three groups: good, mid, bad). After that we divided participants into three groups for each of the nine groups with random number list arbitrarily. There were no significant differences among the body height, body mass and age of the three groups.

All participants were asked to keep a sleep diary throughout the 30 days of the intervention period, which was October-November in 2010. The sleep diary involved the question, “How was the depth of your last night’s sleep?” to which participants answered every morning. The choices for answer were “deep”, “relatively deep”, “relatively shallow” and “shallow”.

Incandescent light bulbs were distributed one by one to the participants in the G3 group, and these participants were asked to install the light bulb in the room in which they slept at night. The G1 and G2 members were asked to switch fluorescent lamps on and the G3 ones were asked to switch incandescent light bulbs on, instead, when they got back to their residences after sunset. After the incandescent light was set, illumination intensity was measured (Table 1). Participants of G2 and G3 were asked to report their breakfast contents, and G3 were also asked to answer the duration of their time spent under incandescent light each day. 63 of 94 (67%) participants answered the first questionnaire and 51 of 63 (81%) kept sleep diaries for 1 month.

**Table 1:** Illumination value (Lux) of all subjects in the third group (G3)

	Standing under the light*						**Sitting as usual**^**#**^					
	**Fluorescent**			**Incandescent**			**Fluorescent**			**Incandescent**		
	**Max**	**Min.**	**Ave**	**Max**	**Min**	**Ave**	**Max**	**Min**	**Ave**	**Max**	**Min**	**Ave**
A	579	571	574	76	74	74	173	158	166	27	25	26
B	845	601	774	42	38	40	143	122	130	30	18	24
C	186	160	170	18	18	18	34	32	33	9	9	9
D	358	344	352	21	15	18	108	100	105	23	9	18
E	1120	1050	1019	32	28	30	89	86	87	18	15	17
F	1042	1013	1025	42	38	40	272	246	257	5	5	5
G	644	572	604	52	50	51	168	103	144	21	19	19
H	3997	2479	2942	70	61	63	284	277	281	39	38	38
I	1200	1002	1106	24	23	23	205	122	163	7	8	7
J	1155	1106	1132	41	41	41	196	184	189	8	6	6
K	657	651	654	72	70	70	65	64	64	20	18	20
L	173	137	148	26	8	19	134	128	130	18	9	18
M	1092	908	1025	377	360	370	597	263	341	50	37	50
N				64	59	61	1			131	114	131
O	505	433	465	160	133	150	231	145	86	39	9	39
P	1307	1193	1264	438	431	434	42	41	41	45	45	45
Q	591	542	572	140	129	136	149	146	147	24	20	24
R	1376	1078	1213	544	483	511	66	49	52	9	8	9
S	1	0	1	346	254	316	1	1	1	24	19	24
T	31	30	30	207	193	202	65	58	61	29	23	29

The surface of illumination meter sensor was put vertically just in front of eyes of participants who are standing* or sitting^#^ and illumination value was measured.

The implementation score was calculated from the sum of days that had “high protein content breakfast” and “exposure to >30 min-exposure to sunlight”. For night exposure to low temperature light, the implemental score was defined as the mean hours (per night for 30 days) when participants were exposed to the low temperature light emitted from the incandescent bulb. After the intervention period for 30 days, the participants were asked to mark the scores on “To what extent do you satisfy on your own carrying out each of the two (G2) or three (G3) contents of intervention as a whole for 30 days, marking as 0-100 points as ‘satisfaction score’ ?” (Table 2).

**Table 2:** Estimates of the extent to which subjects in groups G2 and G3 carried out the intervention

**Question: On a scale of 0 to 100, how would you estimate your confidence in your response? The question is “To what extent did you carry out this intervention program during this one month intervention period?**	
1. Estimate for the whole protocol. (G2, G3)	**score /100**
2. Estimate for “taking protein-rich and Vitamin B6-rich foods at breakfast”. (G2, G3)	**score /100**
3. Estimate for “exposure to sunlight after the breakfast”. (G2, G3)	**score /100**
4. Estimate for “exposure to low color temperature light emitted from incandescent bulbs at night". (G3)	**score /100**

The salivary melatonin was measured of 10 subjects which were randomly selected from each group because of financial limitation for the chemical analysis (30 participants in total) three times: the day before the start of intervention, at the mid-point (two weeks past in the intervention) and the day before the last day of the intervention. Participants were asked to extract their own saliva at around 23:00 and keep it in a freezer. They turned off the lights when they went to bed (ranging from 23:00 to 2:00).

The saliva samples were collected around 23:00 with cylindrical cotton (1 cm diameter, 3 cm long) which was put under the tongue for 3 min. The saliva samples were kept frozen at −25°C until analysis for 1 or 2 weeks. After centrifugation (1000 × g for 5 min), melatonin concentrations in the saliva samples were determined using an ELISA kit (Direct Saliva Melatonin ELISA, Bulmann, Switzerland).

For the statistical analysis, the “implementation rate” was defined as how many days participants had a protein-rich food (1 point) and Vitamin B6-rich food (1 point) at breakfast and, further, exposed to sunlight for more than 30 min after breakfast (1 point). Participants reported how many minutes they were exposed to low-color temperature lights during the 30 intervention days. The 30-day-long intervention period was divided into 3 parts (FWP: First week period, MP: Medium period of 16 days, LWP: Last week period). The “high implementation group” was defined as 50% participants who marked higher implementation rate in both breakfast contents and exposure to sunlight after breakfast (G2 and G3) and also were exposed to longer hours when they were exposed to the low temperature lights each night (G3). The other 50% participants group was defined as “the low implementation group”.

The software used for statistical analysis was SPSS 12.0 J for Windows (SPSS Inc., Chicago, IL, USA). χ^2^-test was used for categorized variables and Mann-Whitney U-tests was used for ranked variables. Pearson’s correlation analysis was performed to test the relationship between two numerical variables.

Before the beginning of the study, participants received a full explanation with the code of the guideline for a study targeting humans [24], including that the results of the study would be used only for academic purposes, and all participants completely agreed to participate in the study.

## Results

### Sleep diary data and salivary melatonin concentration during the 30 days of the intervention

**Figure 2:**
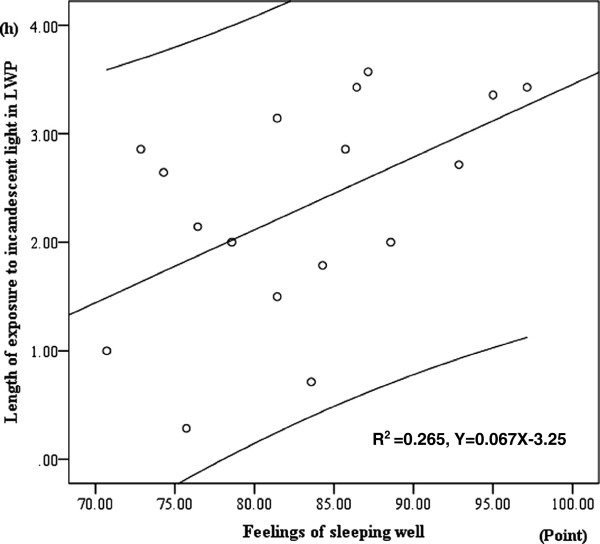
**Positive correlation between hours when subjects were exposed to incandescent light at night and the index of feeling of sleeping well in Last Week Period.** Upper and lower lines of linear regression line show 95% confidence estimate.

There was significant positive correlation between hours spent under incandescent light at night and the feeling of sleeping well in Last Week Period (LWP) (Pearson’s correlation test: r^2^ = 0.265, p = 0.034) (Figure 2).

**Figure 3:**
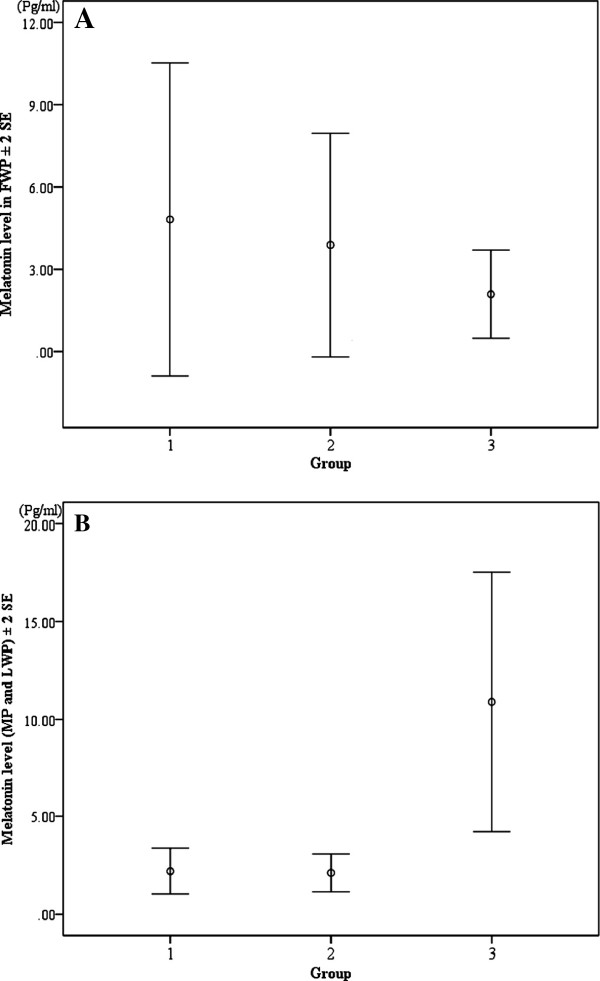
**Comparison of salivary melatonin concentration among the three groups.** Group 1: no intervention; Group 2: Recommendation of high protein breakfast and exposure to sunlight; Group 3: Same as Group 2 plus the recommendation of exposure to low color temperature light emitted from an incandescent light bulb. **A**: Melatonin level in the saliva collected on the day just before the intervention (Kruskal Wallis test: χ^2^-value = 0.92, df = 2, p = 0.63); **B**: Melatonin in the saliva collected at the mid-point and on the day before the last day of the intervention (Bonferroni multiple comparison test: G1 versus G3, p = 0.018; G2 versus G3, p = 0.011).

The concentration of salivary melatonin shown by the participants of G3 was significantly higher than that of G1 and G2 in the mid-point and the day before the last day of the intervention (Bonferroni multiple comparison test: G1 versus G3, p = 0.018; G2 versus G3, p = 0.011), whereas there were no significant differences among the three groups on the day just before the start of the intervention (Kruskal Wallis test: χ^2^-value = 0.92, df = 2, p = 0.63) (Figure 3).

**Figure 4:**
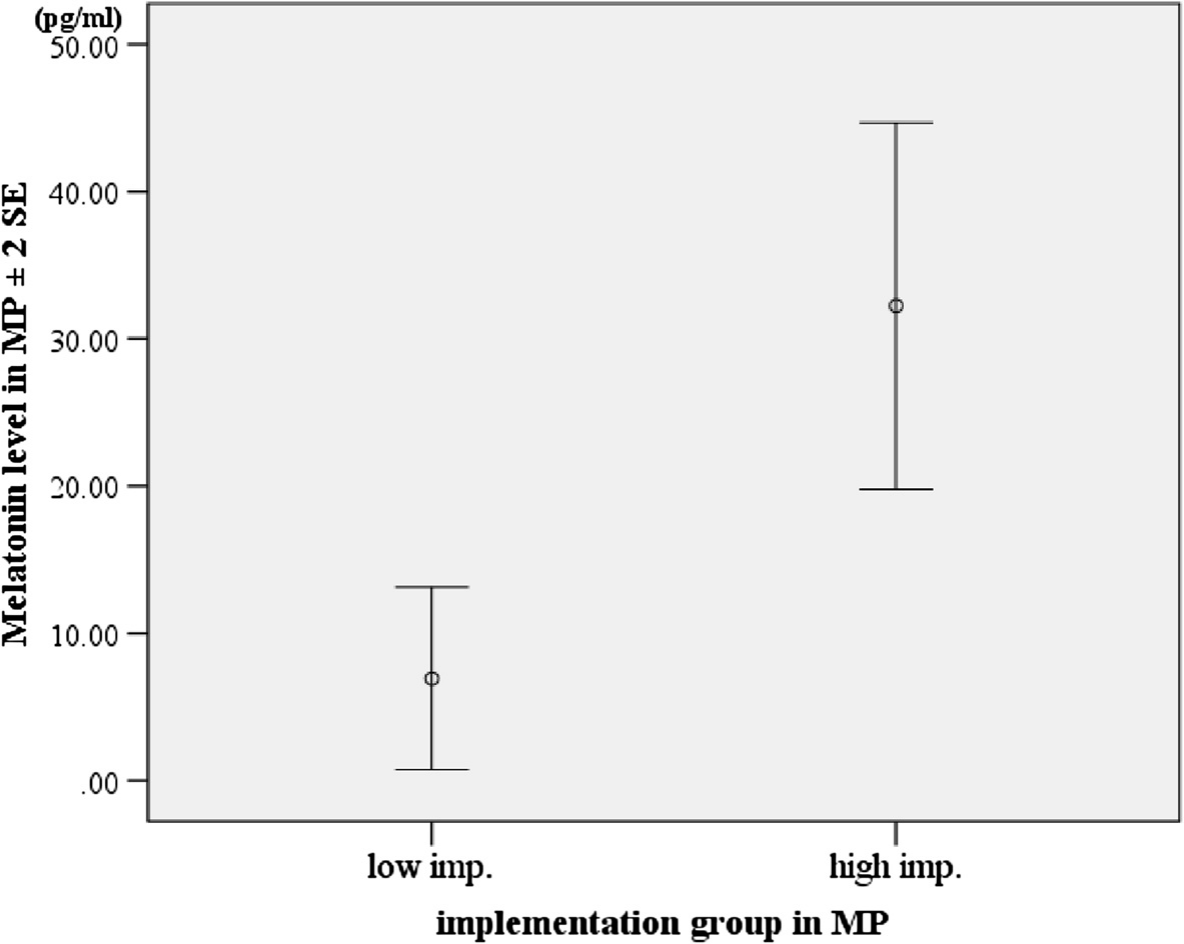
**Comparison of melatonin level between “high implementation group” and “low implementation group” of G3 participants.** See the text for details.

**Figure 5:**
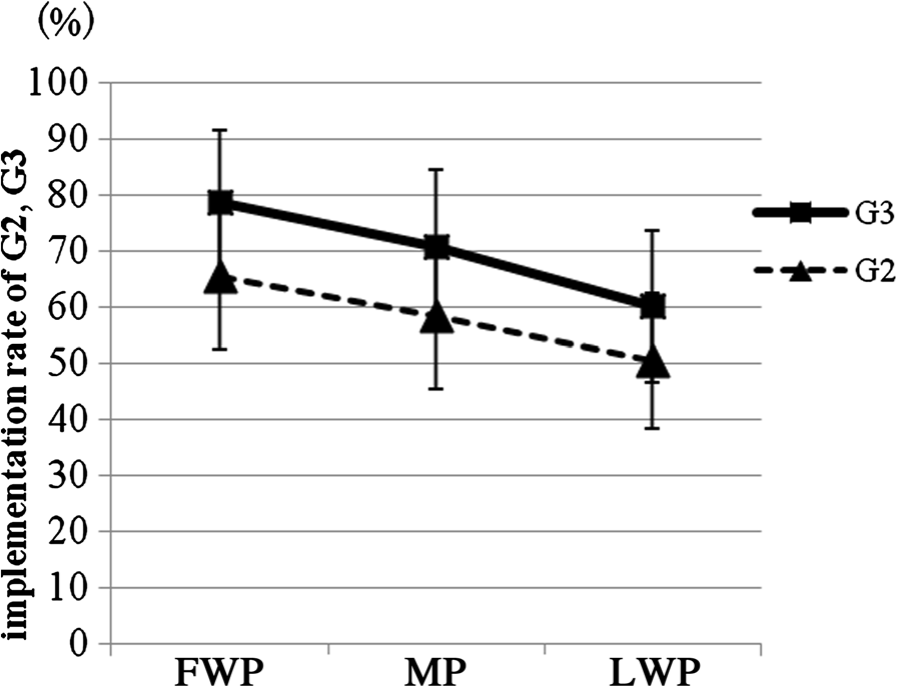
Comparison of how many days students followed the recommendations during the 30-day-long intervention between two groups in the first week period (FWP), medium period of 2 weeks (MP) and the last week period (LWP).

The “high implementation group” tended to show a higher concentration of salivary melatonin in MP than the “low implementation group” did in G3 (Mann-Whitney U-test: z = -2.000, p = 0.071) (Figure 4). Participants of G3 tended to follow the morning intervention recommendations (high protein breakfast and sunlight exposure) on more days than G2 participants did (Mann-Whitney U-test: FWP; z = -1.952, p = 0.053, MP; z = -1.628, p = 0.105, LWP; z = 1.253, p = 0.221) (Figure 5). The implementation rate in FWP tended to be higher than in MP (Wilcoxon’s signed rank sum test,: G2:, z = -1.851, p = 0.064; G3:, z = -1.914, p = 0.056) and LWP (G2:, z = -2.298, p = 0.022; G3:, z = -2.898, p = 0.004). The implementation rate in MP tended to be also higher than in LWP in G2 and G3 (G2:, z = -1.681, p = 0.093; G3:, z = -2.533, p = 0.011).

### Several parameters before and after the intervention period

There was a significantly positive correlation between the implementation satisfaction index (Maximum score: 100, Table 2) and the regularity of time to take breakfast and supper (Kendall tau-b test: breakfast, r = 0.058, p = 0.038; supper, r^2^ = 0.057 p = 0.036).

**Figure 6:**
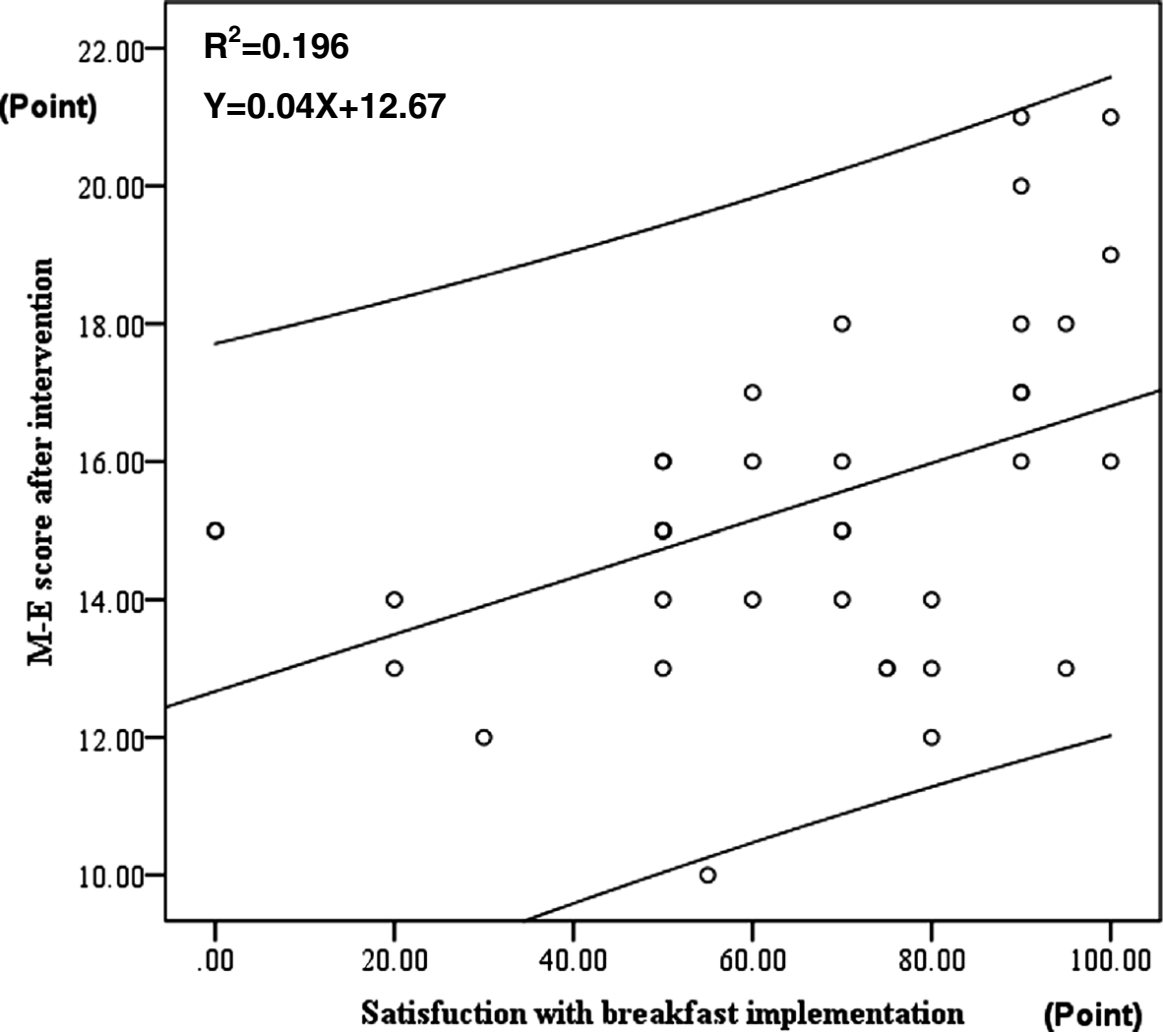
**Positive correlation between M-E scores and the index of satisfaction on the implementation of the intervention on breakfast with high protein foods.** Upper and lower lines of linear regression line show 95% confidence estimate.

There was a significant positive correlation between the index of how many days among the 30 days subjects were satisfied in their own implementation of the intervention (of having a breakfast that includes high protein foods) and M-E scores one month after the intervention (higher scores showing morning-type) (Pearson’s correlation test: r^2^ = 0.195, p = 0.006) (Figure 6).

There was a significant positive correlation between the number of nights when participants were exposed to incandescent light during the month-long intervention and the regularity index of meal time, not only for breakfast, but also for lunch and supper, just after the intervention (Kendall tau b-test: r = -0.574, p = 0.007, r^2^ = 0.146, p = 0.084, r^2^ = 0.215, p = 0.029). Participants who ate breakfast more frequently for one month after the intervention showed a lower frequency of having late night snacks during that month (Kendall tau-b test: r^2^ = -0.142, p = 0.003) than those who ate breakfast less frequently.

**Figure 7:**
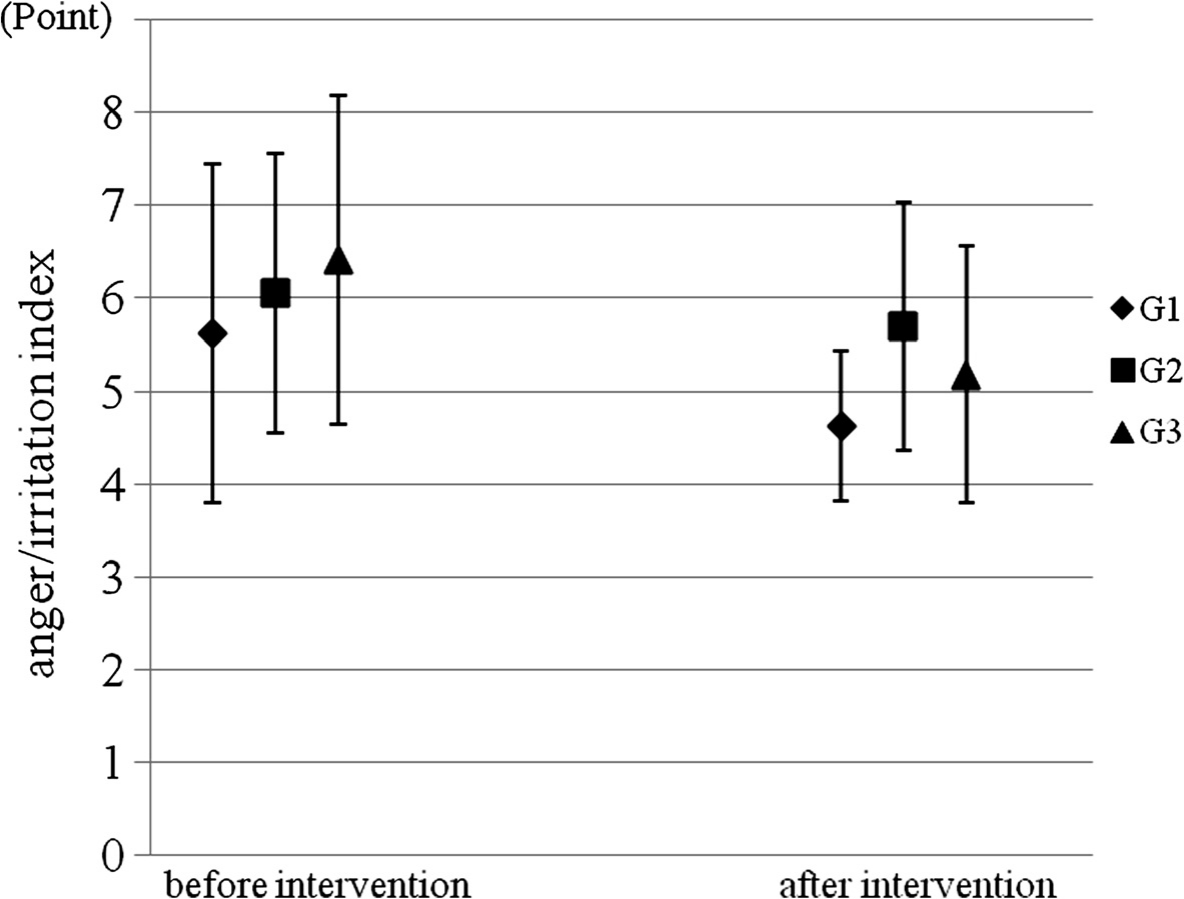
Comparison of the anger/irritation index before and after the intervention.

The participants in G3 after the intervention showed a lower anger/irritation index than before the intervention (Wilcoxon’s signed rank sum test: z = -3.072, p = 0.002), whereas there was only the tendency towards reduced irritation in G1 (z = -1.786, p = 0.074) and no differences were seen in G2 (z = -0.954, p = 0.340) (Figure 7). Two components of the anger/irritation index, the frequency to be irritated and the frequency to become angry due to small trigger, were also reduced after the intervention in comparison with before the intervention in G3 (Wilcoxon’s signed rank sum test: irritation, z = -2.496, p = 0.013; anger;, z = 2.714, p = 0.007).

## Discussion

This study showed that a triple intervention concerning breakfast content, sunlight exposure after breakfast and exposure to low temperature light emitted from incandescent bulbs is a powerful method for inducing secretion of high amounts of melatonin by the pineal gland in human adults. Underlying mechanisms can be hypothesized to consist of two components. The first is that serotonin synthesis from tryptophan taken at breakfast may be enhanced by the exposure to sunlight just after taking breakfast. The second is that the high potential of melatonin synthesis based on the high serotonin synthesis in the pineal during daytime might be available due to the night exposure to the “low temperature light” emitted from incandescent bulbs. Although many reports have shown that melatonin secretion is suppressed by light emitted from fluorescent lamps including short wave length (with around 460 nm of wave length) components [25-27], and especially short wave length light [27-30], this study newly implies that the combined behaviors of modifying breakfast content, receiving sunlight exposure and receiving exposure to low color temperature lighting at night can facilitate achievement of high plasma melatonin at night in humans.

Melatonin, a hormone secreted from the pineal gland, causes the core body temperature to decrease and induces sleep [31-33]. High plasma melatonin levels at night may play an important role in sleep onset and sleep quality [34]. In this study, the longer time participants spent under incandescent lights at night, the significantly higher scores they marked to feel deep sleep. This better sleep quality might be due to high plasma melatonin levels.

This intervention study supports the hypothesis that the triple intervention of having sources of tryptophan and vitamin B6 at breakfast, following up breakfast with exposure to sunlight and the exposure to low temperature lights as night lighting can stimulate the synthesis of serotonin and succeeding melatonin synthesis at night and that these hormones work as natural anti-depression drugs and/or natural sleeping pills and make students more-morning typed and improve their mental health.

A limitation of this study as a “field intervention experiment” is that we did not include a control group with low-tryptophan breakfast, sunlight exposure, and exposure to low temperature light to find out the importance of the intake of tryptophan at breakfast for the mechanism of tryptophan-serotonin-melatonin pathway more clearly. This study was not a “physiological experiment” to set up several experimental groups and control all the environmental conditions, and such experiment remains to be conducted in the future. Another limitation of this study is that it was performed only with men, whereas the inclusion of participants from a female sports club could add important data on gender differences in response to breakfast modulation and the change in lighting at night.

### Consent

Written informed consent was obtained from the participants for publication of this report and any accompanying images.

## Competing interest

The authors declare that they have no competing interests.

## Authors’ contributions

KW: planned the study, conducted the intervention experiments, analyzed data, participated in the discussion of the results, and drafted the manuscript. SY: conducted the intervention experiments, analyzed data, and participated in the discussion of the results. OA: participated in the discussion of the results. MK: participated in the discussion of the results. TN: planned the study and participated in the discussion of the results. MN: planned the study, analyzed data on nutrition and participated in the discussion of the results. HT: planned the study, analyzed data and participated in the discussion of the results. TH: supervised the project, participated in the discussion of the results, and edited the manuscript. All authors read and approved the final manuscript.
